# Processing and Profile Control of Microhole Array for PDMS Mask with Femtosecond Laser

**DOI:** 10.3390/mi13020340

**Published:** 2022-02-21

**Authors:** Xifang Zhang, Zhenqiang Yao, Zhibao Hou, Jiacheng Song

**Affiliations:** 1 School of Mechanical Engineering, Shanghai Jiao Tong University, Shanghai 200240, China; xfzhang0103@sjtu.edu.cn (X.Z.); houzhb@sjtu.edu.cn (Z.H.); sjc9755@163.com (J.S.); 2 College of Mechanical Engineering, Suzhou University of Science and Technology, Suzhou 215009, China; 3 State Key Laboratory of Mechanical System and vibration, Shanghai Jiao Tong University, Shanghai 200240, China

**Keywords:** polydimethylsiloxane (PDMS), femtosecond laser, laser ablation threshold, micro-through-hole, cone geometry, profile quality

## Abstract

Polydimethylsiloxane (PDMS) is hailed as one of the foundational materials that have been applied to different products in various fields because of its chemical resistance, low cost, excellent flexibility, and high molding capability. With the aim to achieve surface texture with high efficiency by means of electrochemical micromachining with PDMS mask, a femtosecond laser is utilized to process a precision array of micro-through-holes on PDMS films as the molds. The ablation process of PDMS with a femtosecond laser was investigated via numerical simulation verified with experiments indicating a laser energy density of 4.865 mJ/mm^2^ as the ablation threshold of PDMS with the melting temperature of 930 K. The spiral scanning path with optimized radial offset was developed to ablate materials from the PDMS film to form the laminated profiles, and a tapered through hole was then formed with multilayer scanning. The profile dimension and accuracy were examined as control targets in terms of laser pulse energy and scanning speed, showing that a 12 μJ femtosecond laser pulse energy and 1000 mm/s scanning speed could bring about a nearly circular laminating profile with expected smaller exit diameter than the entry diameter. All the cross-section diameters of the microcone decreased with the increase of laser scanning speed, while the taper increased gradually and then saturated around a laser scanning speed of 800 mm/s due to the energy absorption resulting in smaller ablation in diameter and depth.

## 1. Introduction

Polydimethylsiloxane (PDMS) microstructured components have been widely used in many fields, especially in microfluidics, optical microelectromechanical systems, micro fabrication techniques, and biomedical applications due to PDMS’s chemical resistance, low cost, low interfacial free energy, excellent flexibility, and high molding capability [1,2,3,4]. A novel hydrophobic and superhydrophobic surface prepared with microhole-arrayed PDMS was able to realize an underwater bubble unidirectional self-transportation in the direction of both buoyancy and antibuoyance [5]. The experiments verified that PDMS microchannels with a high aspect ratio of 10 and depth of 200 μm could reduce the interfacial adhesion in soft lithography [6]. Metal-based PDMS composites have been used in sensing and actuating applications where electrical conductivity and mechanical flexibility are of interest [7]. PDMS is a silicone-based synthetic material used in various biomedical applications and the investigations exhibited that surface modification of PDMS-based microfluidic devices with collagen using polydopamine as a spacer to enhance primary human bronchial epithelial cell adhesion [8].

The fabrication of PDMS in microfluidics has attracted worldwide attention owing to the key applications of PDMS in some of the most important domains of science and engineering: chemical and material, mechanical and fabrication, biological, biomedical, biomimetics, and sensors and actuators [9,10,11,12]. It is well known that soft lithography is the most commonly method for generating PDMS microstructured components. It is a group of micro-/nanofabrication techniques that use elastomeric polymer materials to replicate and transfer micro-/nanostructures from original masters [13,14,15]. Experiments verified that the simple SU-8 coating protocol allows for the successful curing of PDMS from any 3D-printed mold, leading to increased rapid manufacturing of complex channels [16]. A direct mechanical micromilling process using cryogenic cooling was explored to cut PDMS fluidic chips for reducing development time and prototyping costs [17,18]. A cryogenic abrasive air jet machining (CAAJM) experimental system was designed for improving the processing quality of direct-write microchannels on PDMS, where the high quality surface was achieved because of CAAJM could realize the erosion processing or the elastomer PDMS material at a vertical angle [19]. 

PDMS with through-holes was first employed as a mask in through mask electrochemical micromachining (TMEMM) for obtaining microstructures, and the PDMS mask was fabricated through two steps of photolithography and molding, where the photolithography process for achieving the SU-8 mold with the micropillar array was complex, time-consuming, and expensive, including spin coating, prebaking, exposure, development, and postbaking [20,21]. It is necessary to prepare through-holes on PDMS mask with high convenience, efficiency, and machining accuracy for surface textures machined in TMEMM. Since the erosion along radial direction during electrochemical machining process will cause an oversize effect in diameter, the PDMS film masks with tapered through holes are preferred.

The application of laser for the manufacture of metallic and polymer materials has become increasingly popular in recent years. Laser ablation is a noncontact process and can achieve high-resolution geometries. Three main types of lasers can be used, including continuous wave, pulsed (nanosecond), or ultrafast pulsed (femtosecond) [22]. The femtosecond laser, with extremely short pulse width, ultrahigh instantaneous power, micro-/nanoprocessing accuracy, almost nonexistent thermal effects to the adjacent materials around the ablation spot, and three-dimensional direct writing nature, is an ideal processing technology for achieving through-holes on PDMS mask [23]. The investigations exhibited that a micro-/nanoscale hierarchical rough structure was formed on PDMS surface by one-step femtosecond laser ablation [24]. A rapid prototyping technique for 3D PDMS microfluidic devices with a low surface roughness of 0.361 nm was demonstrated utilizing a red femtosecond laser to produce a metallic mold, which was then directly used for the replica molding of PDMS [25]. PDMS-based volume Bragg gratings (VBGs) were successfully fabricated by stitching of the femtosecond laser filament, and the diffraction efficiency has been experimentally investigated by varying the scanning speed, energy, number of layers, and spacing between layers [26]. Experimental investigations showed that direct writing of copper (Cu) patterns on PDMS using femtosecond laser could induce thermochemical reduction of the glyoxylic acid copper (GACu) complex [27]. 

The present work focuses on the fabrication and profile control of the microhole array by means of femtosecond laser for PDMS mask so as to obtain micro structures and surface morphology with high efficiency and high quality in electrochemical micromachining. The PDMS films were prepared using a vacuum-aided process, in which the cured PDMS film could be smoothly peeled off from the molds. The numerical simulations and experimental validations were followed to investigate the laser ablation threshold of PDMS films and the effects of laser pulse energy and laser scanning speed on material removal. PDMS with through holes as a mask were processed by means of ultrafast pulsed femtosecond laser with the spiral scanning path under radial offsets were investigated. The influence of processing parameters on the cone profiles and dimensions of PDMS micro-through-holes were examined for controlling the profile and the size of the exit holes.

## 2. Modeling and Simulation of the Ablation Threshold

The solid heat transfer model in COMSOL Multiphysics package was applied to simulate the ablation threshold of PDMS material. The numerical simulation is carried out based on the following hypotheses [28]:

(a) The material is homogeneous and isotropic;
(b) The lifetime of induced plasma (~100 ps) is much longer than the laser pulse;
(c) The diffusion of plasma is neglected in the timescale of the laser pulse;
(d) In the range of interests, the laser beam can be regarded as collimated.

The femtosecond laser beam is assumed to obey Gaussian distribution, and the laser intensity *I* on the focal plane can be expressed as follows [28]:

(1) $$I(t,r,0)=\frac{2F}{\sqrt{\pi /\ln2\tau_{p}}}(1-R)\exp(-\frac{r^{2}}{r_{0}^{2}}-(4\ln2)\frac{t^{2}}{\tau_{p}^{2}})$$

(2) $$F=\frac{P_{0}}{\pi r_{0}^{2}}$$

where *t* is the time, *F* is the laser energy density (also known as laser fluence), *τ_p_* is the pulse duration of femtosecond laser, *P*_0_ is the single pulse laser energy, *r* is the distance along the radius direction, and *r*_0_ is the laser spot radius at focal plane. *R* is the surface reflectivity of PDMS and nearly to 0 because of the PDMS is a black, nontransparent material.

The heat transfer during PDMS ablation process with femtosecond laser can be expressed as

(3) $$\rho C_{p}{\frac{\partial T}{\partial t}}_{}+\rho Cpu\cdot \nabla T+\nabla \cdot q=Q$$

(4) $$q=-k\nabla T_{\;}$$

where *ρ* presents the material density, *C_p_* is the specific heat capacity of material, *k* is the thermal conductivity of material, *q* indicates the heat flux, *T* is the temperature of material surface, *t* is the processing time, *Q* is the total heat source energy, and *u* denotes the convection speed. 

**Figure 1:**
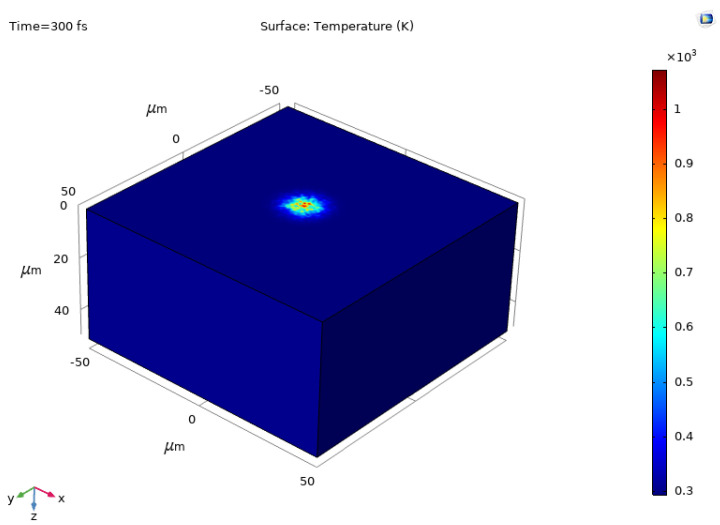
Surface temperature distribution in PDMS during laser ablation with threshold energy.

The model size is selected as 0.1 mm × 0.1 mm × 0.05 mm based on the laser spot size as shown in Figure 1, where a fixed Gaussian distribution laser as a heat source is loaded on the upper surface of the model and the rest of the surfaces are insulated. The model is discretized into free tetrahedron meshes for simulation. The values of simulation parameters are listed in Table 1.

Figure 1 shows the result of numerical simulation of ablation temperature on PDMS material surface. It can be noticed that the ablation temperature reaches 930 K with the ablation threshold at laser energy density of 4.865 mJ/mm^2^, which is consistent with the melting point of the PDMS material. Furthermore, the temperature in the center of the laser spot is higher than that at the edge, which implies that the material starts to be ablated from the center to edge area, where ablations occur when the accumulated heat fluence reaches the melting point of the material [29]. 

## 3. Material Preparation and Process Methodology

### 3.1. Material Preparation of PDMS Film

**Figure 2:**
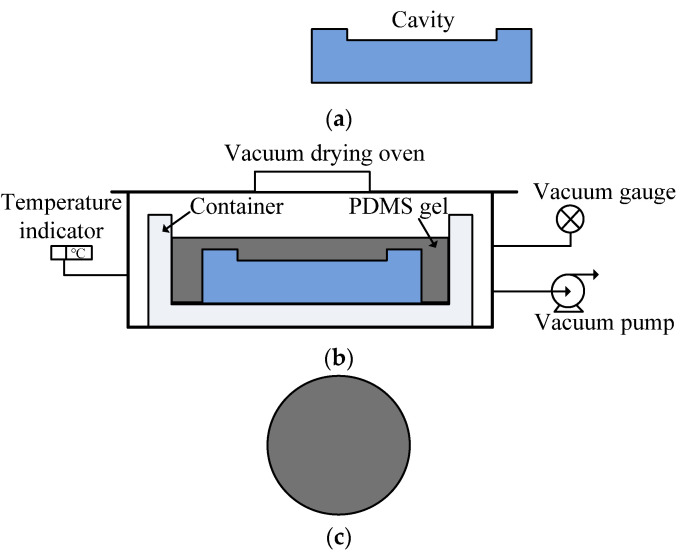
Schematic diagram of preparation process of PDMS film: (**a**) The stainless steel mold with cavity is prepared; (**b**) PDMS gel is filled into the mold in the vacuum chamber; (**c**) PDMS film layer is cured and removed from the mold.

PDMS prepolymer is an odorless, nonvolatile viscous liquid at room temperature and is cured by the catalytic reaction of a cross-linking agent. PDMS gel (Sylgard 170, Dow Corning Corp, Midland, MI USA) was employed to prepare the PDMS film using a vacuum-aided process. The fabrication process of PDMS film is schematically as shown in Figure 2. First, the stainless steel mold was milled and polished to form a cavity with a depth of 0.15 mm and diameter of 80 mm, which was placed in a container. Then, the PDMS gel composed of a mixture of PDMS base and curing agent at the suggested ratio of 1:1 was poured into the container [21]. After that, the container with the PDMS gel was placed in the vacuum drying oven to remove the air for 10 min, so that the cavity mold was filled with the mixed PDMS gel. Subsequently, the PDMS gel is solidified in an oven at 70 °C for 30 min. Finally, the cured PDMS film layer was smoothly peeled off from the mold at the room temperature.

The study of the influence of PDMS mask film thickness on electrochemical micromachining quality has revealed that high quality microdimples can be achieved in microfabrication with through mask electrochemical micromachining (TMEMM). The uniform current density distribution can be obtained over the through holes when the thickness of PDMS mask is larger than 100 μm, resulting in the microdimple prepared with a flat bottom [30]. The research achievements supplied a criterion on selection of the PDMS film thickness for femtosecond-laser-assisted mask processing.

### 3.2. Fabrication of Micro-Holes on PDMS with Femtosecond Laser Ablation 

The femtosecond laser technique, as an efficient and convenient machining method, is more environment-friendly and controllable, which has attracted more attention in microfabrication due to its extremely narrow pulse width, ultrahigh instantaneous power density, high processing accuracy, and almost nonexistent thermal effects to the surrounding materials. Compared with the long pulse width laser, the processing characteristics of femtosecond laser are closer to that of cold machining, where the damage on the surface and chemical properties of the material can be ignored. The femtosecond laser has great application for processing a wide range of materials including metals, alloys, ceramics, and polymers [31]. 

**Figure 3:**
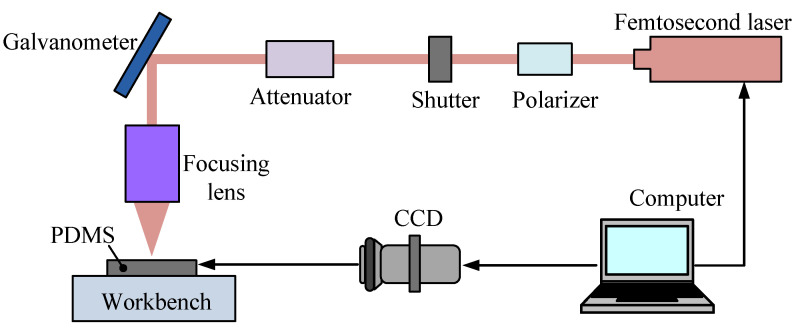
Schematic diagram of the experimental setup for femtosecond laser ablation of PDMS.

The femtosecond laser ablation system is applied to prepare the microhole array on PDMS film. As shown in Figure 3, the experimental setup consists of a femtosecond laser (Carbide-40 W, Light Conversion, Vilnius, Lithuania) with 218 fs pulse duration, 1028 nm wavelength, 1 MHz base pulse frequency, and 400 μJ maximum pulse energy. The number of laser pulses reaching the target material is selected by means of an electromechanical shutter. A variable energy selector, formed by a half wave plate and a polarizer, is exploited to control the laser pulse energy. The energy of the incident beam is controlled by the attenuator, which is set between the laser device and the galvanometer. The PDMS film is placed on the workbench, and a charge coupled device (CCD) is connected to the computer to monitor the PDMS surface.

The detailed processing parameters for femtosecond laser ablation through holes on PDMS film are listed in Table 2. The femtosecond laser with laser spot diameter of 12 μm and laser pulse frequency of 100 kHz was used to process the PDMS film with thickness of 150 μm. 

**Figure 4:**
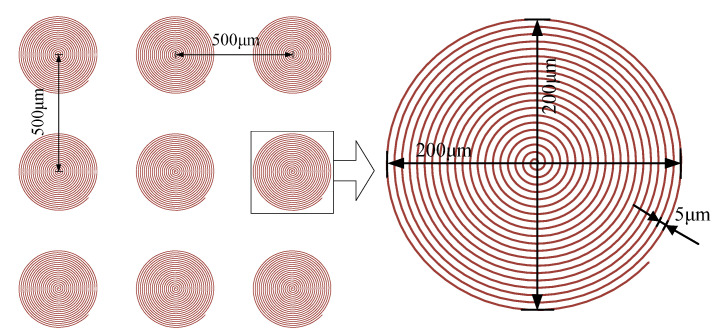
Schematic diagram of the femtosecond laser spiral scanning path.

**Figure 5:**
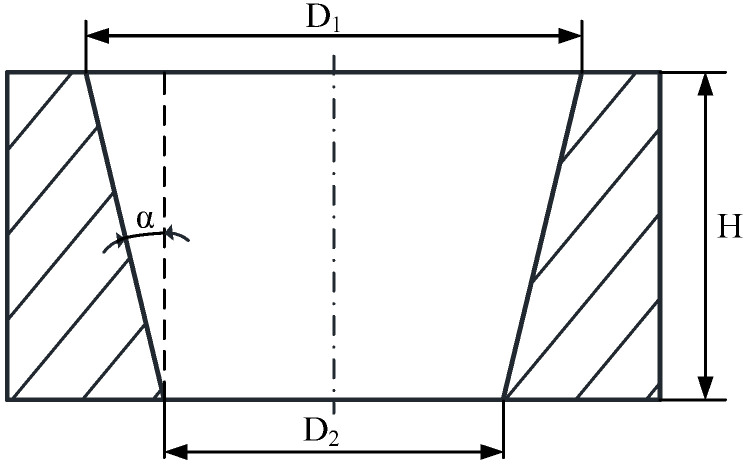
Schematic diagram of the through-hole taper.

Each through-hole with a diameter of 200 μm was processed using laser spiral scanning path with a radial pitch of 5 μm as shown in Figure 4. The through-hole was finished after 20 times multiple scanning to achieve a taper shaped cone as shown in Figure 5. The 3 × 3 array of through-holes with both the row and column offset to be 500 μm were directly obtained without the motion of worktable thanks to the galvanometer with a large scanning area of 60 mm in diameter. The lateral feeding system supporting PDMS films would be used to prepare arrays with large scale offset or over large area.

With the parameters of the laser processing system, the Rayleigh length determined by the laser wavelength and Gaussian beam radius [32] can be predicted as 110 μm, hence the laser focal length can be considered constant within the PDMS thickness of 220 μm, which means that the spiral scanning path has almost no influence on the through hole quality within the PDMS thickness of 220 μm. A vertical feeding system would be necessary to adjust the laser focal length between the galvanometer and the workbench if thicker PDMS masks are to be processed. 

The pulse energy associated with the laser ablation threshold of PDMS was explored from 0.5 μJ to 12 μJ, and the effects of laser scanning speed on dimensions of entry and exit holes and through hole taper have been investigated from 200 mm/s to 1000 mm/s. Three experiments were conducted for each group of femtosecond laser parameters to confirm the reproducibility and repeatability. The geometric profiles of the microholes were measured using a microscope (BX51, Olympus, Tokyo, Japan) with a resolution of 373 nm, and the surface morphology of microhole arrayed on PDMS was observed using a scanning electron microscope (SEM, VEGA3 TESCAN, Brno, Czech Republic). The through-hole with a taper shape is closely related with femtosecond laser processing parameters where the entry profile and exit profile were mainly influenced with laser pulse energy and scanning speed as well as the multiple scanning cycles. The taper shape as shown in Figure 5 can be expressed from the geometric relation as follows:

(5) $$\alpha =\tan^{-1}[\frac{D_{1}-D_{2}}{2H}]$$

where *α* is the through-hole taper, *H* represents the thickness of the PDMS film, *D*_1_ denotes the entry diameter, and *D*_2_ denotes the exit diameter as the most important parameters determined with laser pulse energy and scanning speed as well as the multiple scanning cycles.

## 4. Results and Discussion

### 4.1. The Ablation Threshold of Laser Pulse Energy on PDMS Film 

**Figure 6:**
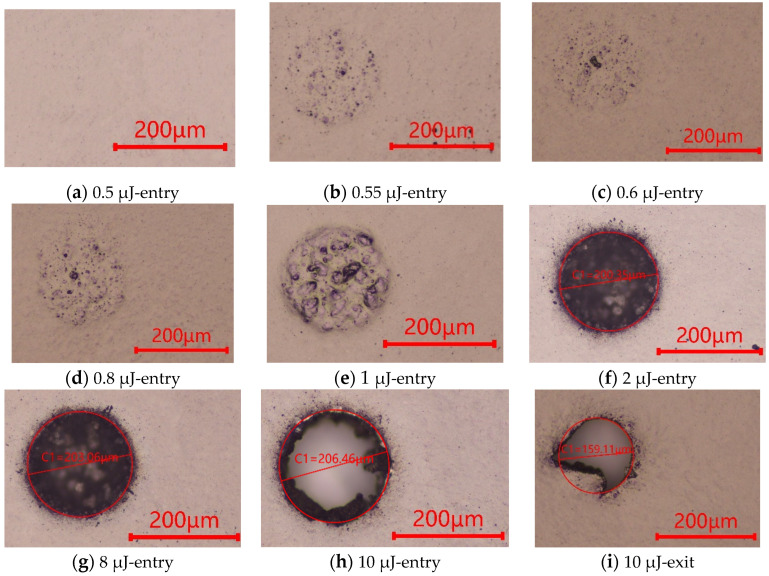
Geometric profiles of the microholes generated with different laser pulse energy; (**a**) entry hole at the energy of 0.5 μJ; (**b**) entry hole at the energy of 0.55 μJ; (**c**) entry hole at the energy of 0.6 μJ; (**d**) entry hole at the energy of 0.8 μJ; (**e**) entry hole at the energy of 1 μJ; (**f**) entry hole at the energy of 2 μJ; (**g**) entry hole at the energy of 8 μJ; (**h**) entry hole at the energy of 10 μJ; (**i**) exit hole at the energy of 10 μJ.

To explore the correlation between femtosecond laser pulse energy and laser ablation threshold of the PDMS film, micro-through-holes were fabricated with laser pulse energy in the range of 0.5–12 μJ when the same laser scanning speed of 1000 mm/s was adopted. The geometric profiles of micro-through-holes obtained with different laser pulse energy are indicated in Figure 6. It can be clearly observed that the PDMS material shows no signs of ablation at the femtosecond laser energy of 0.5 μJ, while the ablation marks occurred when the laser energy is higher than 0.55 μJ, indicating the ablation threshold of the femtosecond laser on PDMS film. The experimental verification of the PDMS ablation threshold at a laser energy density of 4.865 mJ/mm^2^ is consistent with that of the numerical simulation.

Processing of micro-through-holes with different laser pulse energies at the constant scanning speed of 1000 mm/s was investigated to check the ablation profile results. Only pitting ablations occurred as islands on the PDMS material entry surface with the laser energy ranging from 0.55 μJ to 1 μJ. The nonuniformity of PDMS film in absorption of laser energy in addition to the nonuniform topography to reflect the laser beam should be responsible for the noncontinuous ablation over the spiral path. The blind holes could be obtained in the PDMS film using femtosecond laser pulse energy ranging from 2 μJ to 8 μJ, indicating that the laser energy is high enough to ablate PDMS film around the spiral path to achieve a high-quality profile, but not sufficient to ablate all the material through the whole depth. The fixed scanning cycles together with lower laser pulse energy in the processing accounts for the results. The processing results suggest that a moderate laser pulse energy ranging from 2 μJ to 8 μJ with more scanning cycles may be a possible strategy to get high quality micro-though-holes on PDMS films. The through-holes can be achieved with fixed scanning cycles of 20 when the laser energy is up to 10 μJ where the burs can be observed at the exit surface. The Gaussian energy distribution near the cone profile account for the existence of the fillet. When the laser pulse energy is further increased to 12 μJ, a high-quality exit profile can be achieved.

### 4.2. Effect of Laser Scanning Speed on the Micro-through-Holes on PDMS

**Figure 7:**
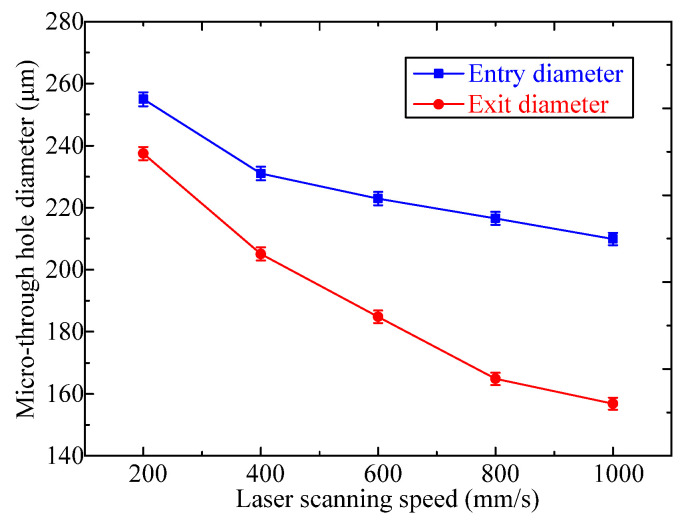
Effect of laser scanning speed on mean diameters of entry and exit through holes with the femtosecond laser pulse energy of 12 μJ.

Laser scanning speed is another parameter that is correlated with the micro-through-holes fabricated with the femtosecond laser process. The mean entry and exit diameters over the micro-through-hole arrays processed with different laser scanning speed and the constant laser pulse energy of 12 μJ were examined, where the sizes of both the entry diameter and exit diameter decrease with the increase of laser scanning speed, as shown in Figure 7. When the laser scanning speed varied from 200 mm/s to 1000 mm/s, the entry diameter decreased from 254.9 μm to 209.8 μm, with a shrink rate of 17%, and the exit diameter decreased from 237.4 to 156.7 μm, with a shrink rate of 34%, respectively. For the femtosecond laser with a pulse frequency of 100 kHz, when the laser scanning speed was increased, the offset of the adjacent laser spots got far away, resulting in less laser ablation from the PDMS film. Therefore, both entry and exit diameters of through holes decreased with the increasing of laser scanning speed. 

**Figure 8:**
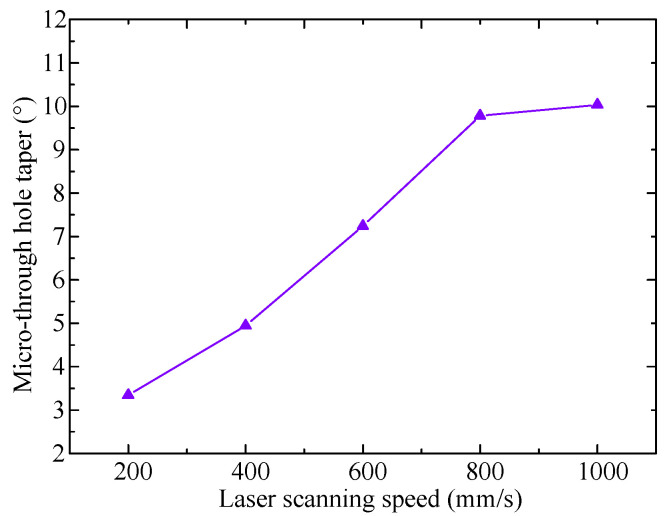
Effect of laser scanning speed on taper of micro-through-holes with the pulse energy of 12 μJ.

The influence of laser scanning speed on the taper of the through holes can be observed in Figure 8, where the taper increases from 3.3 degree to 10 degree at the laser scanning speed ranging from 200 mm/s to 800 mm/s and saturated from 800 mm/s to 1000 mm/s. The micro-through-holes with cone like shape are attributed to both the Gaussian distribution of laser energy and the decreased ablation rate with increased scanning speed. According to the spiral laser scanning regime with a radius offset of 5 μm and laser spot diameter of 12 μm, the initial scanning path started from the outer side of the processing area where no spot overlap existed, resulting in the fillets that accumulated to from a slope side wall of the cone layer by layer. The existence of the fillets would decrease the exit diameter at the same time. The ablation rate decreased with increased laser scanning speed bringing about the larger fillet and hence larger cone angle and smaller exit diameter. In addition, the absorbed laser beam energy decays from the focus plane along the direction of the PDMS film depth, which is also responsible for the shrinking from entry to exit hole. 

**Figure 9:**
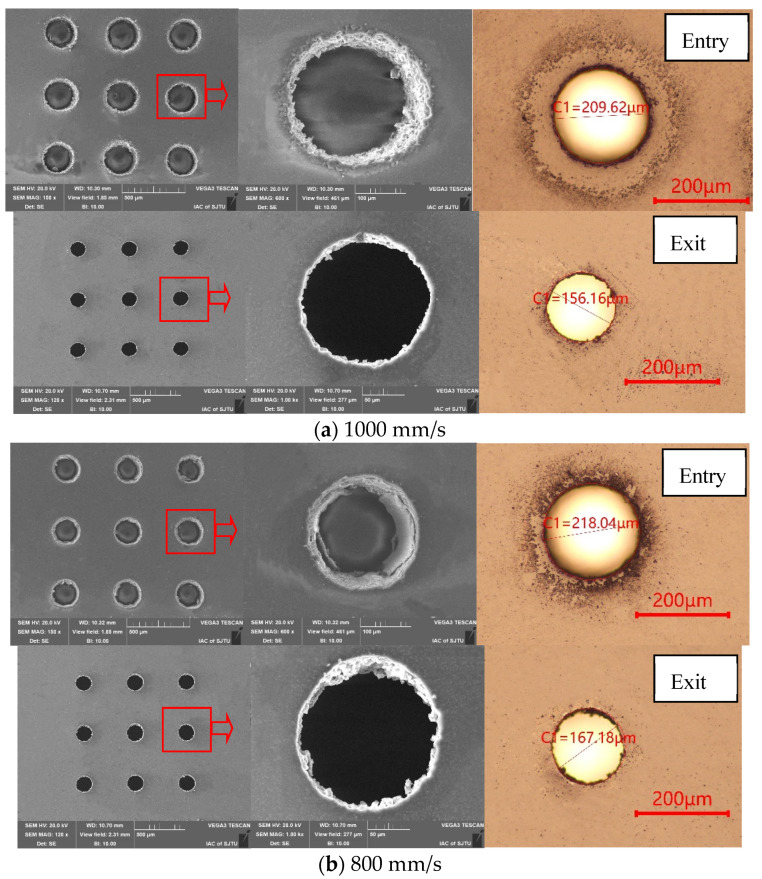

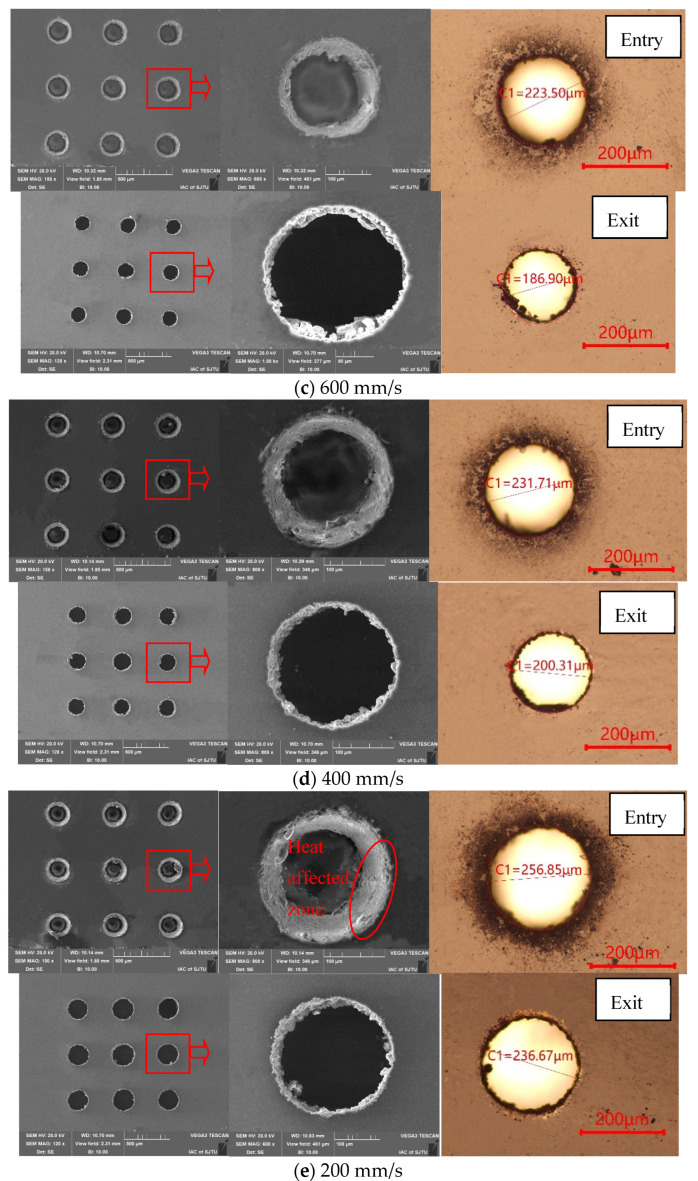
Geometric profiles and SEM images of the microholes generated at different laser scanning speed at the pulse energy of 12 μJ; (**a**) 1000 mm/s; (**b**) 800 mm/s; (**c**) 600 mm/s; (**d**) 400 mm/s; (**e**) 200 mm/s.

The effects of laser scanning speed on profile quality can be observed in Figure 9 when the scanning velocity decreased from 1000 mm/s to 200 mm/s while the laser pulse energy kept steady at 12 μJ. The SEM micrograph and microscope image of micro-through-holes in PDMS film under each laser processing condition were taken from both the entry side and the exit side. With the decreasing of laser scanning speed, the quality of entry profiles deteriorated with a rough entry brim. This is attributed to the pulse-to-pulse distance narrowing and the application of more laser spot overlaps to the outside of the processing area with the laser scanning speed decreased from 1000 mm/s to 200 mm/s at a constant pulse frequency of 100 kHz, resulting in more heat dissipation from the laser spot over the top layer spiral scanning path. Another potential reason is that the reaction force generated by femtosecond laser ablation of molten spatter products is opposite to the laser incident direction, which leads to the rapid accumulation of molten spatter products at the edge of the inlet through hole under the action of vapor viscosity [33]. Each exit profile kept higher quality than entry profile, which is due to the counteracting of more heat dissipation and fillet accumulation at the cross-through circle between the exit plane and the cone. 

**Figure 10:**
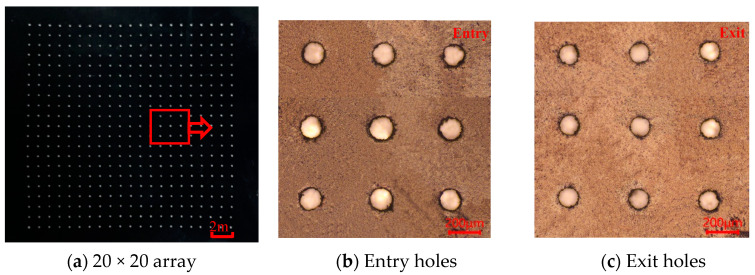
Microscope image of microholes on PDMS generated at laser scanning speed of 1000 mm/s and the pulse energy of 12 μJ; (**a**) 20 × 20 array of microholes; (**b**) 3 × 3 array of entry holes; (**c**) 3 × 3 array of exit holes.

The investigations showed that the micro-through-hole arrayed PDMS mask with better surface quality can be obtained with a femtosecond laser pulse energy of 12 μJ and a spiral laser scanning speed of 1000 mm/s with radius offset of 5 μm and a laser spot diameter of 12 μm, where the 20 × 20 array of through holes were prepared on PDMS mask to achieve large-scale surface texture in electrochemical micromachining for future investigation as shown in Figure 10.

## 5. Conclusions

The fabrication process of micro-through-holes on PDMS film by means of femtosecond laser with spiral scanning path was examined. Based on the numerical simulation and experimental investigation, the following conclusions can be drawn:

The femtosecond laser processing method is capable of fabricating micro-through-holes on PDMS films. PDMS with through-holes has been used as a mask to achieve large-scale surface texture in electrochemical micromachining, which was prepared by means of femtosecond laser with high convenience and efficiency, compared with the complex, time-consuming, and expensive photolithography process. The experimental verification of the PDMS ablation threshold at laser density of 4.865 mJ/mm^2^ is consistent with that of numerical simulation.

Three ablation approaches can be adopted with the increase of laser pulse energy. Only pitting ablations occur like islands on PDMS film entry surface with the laser energy ranging from 0.55 μJ to 1 μJ. The blind holes can be generated in the PDMS film using the laser energy ranging from 2 μJ to 8 μJ. The through-holes can be achieved when the laser energy is up to 10 μJ where the burs can be observed at the exit surface, and a high quality exit profile can be achieved when the laser pulse energy is increased to 12 μJ. 

The laser scanning speed plays a key role in micro-through-hole geometries. The entry diameter and exit diameter decrease with the laser scanning speed increased from 200 mm/s to 1000 mm/s at the same laser pulse energy of 12 μJ, and the hole taper increases from 3.3 degree to 10 degree at the laser scanning speed in the range of 200–1000 mm/s. With the spiral scanning laser processing parameters of 12 μJ laser pulse energy and 800–1000 mm/s scanning speed, a steady taper angel and less variation in entry and exit diameter can be achieved. 

The experiments show that the quality of the entry edge of through holes deteriorates with the lowering of laser scanning speed, and micro-through-holes with better surface quality can be obtained at the laser pulse energy of 12 μJ and the laser scanning speed of 1000 mm/s.

The investigation of laser ablation threshold of PDMS and the effects of laser pulse energy and laser scanning speed on the through holes geometries could provide theoretical value for the selection of laser machining parameters for the fabrication of through-holes and microchannels on different thickness of PDMS. The fabrication of micro-through-holes by means of femtosecond laser with spiral scanning paths could be a potential approach for processing biological samples with the controlling of processing parameters.

## Figures and Tables

**Table 1:** The values of simulation parameters for laser ablation threshold.

Parameter	Value
Material density, *ρ*	950 kg/m^3^
Thermal conductivity of material, *k*	0.2 W/(m·K)
Specific heat capacity of material, *C_p_*	35,000 J/(kg·K)
Laser spot radius, *r*_0_	6 μm
Laser pulse duration, *τ_p_*	218 fs
Laser pulse frequency, *f*	100 kHz
Single pulse laser energy, *P*_0_	0.55 μJ

**Table 2:** The processing parameters for femtosecond laser through-holes on PDMS.

Parameter	Value
Laser wavelength (nm)	1028
Pulse duration (fs)	218
Pulse frequency (kHz)	100
Minimal laser spot diameter (μm)	12
Thickness of PDMS (μm)	150
Laser scanning cycles	20
Laser scanning path	Spiral scanning
Laser scanning speed (mm/s)	200, 400, 600, 800, 1000
Laser pulse energy (μJ)	0.5, 0.55, 0.6, 0.8, 1, 2, 4, 6, 8, 10, 12

## Data Availability

The data that support the findings of this study are available from the corresponding author upon reasonable request.

## References

[1] Wolf M.P., Salieb-Beugelaar G.B., Hunziker P. (2018). PDMS with designer functionalities—Properties, modifications strategies, and applications. Prog. Polym. Sci..

[2] Cao C., Liang F., Liu R., Zhang Y., Zhang W., Zhu T., Yi B., Tang Y., Lai Y. (2020). “PDMS-in-water” emulsion enables mechanochemically robust superhydrophobic surfaces with self-healing nature. Nanoscale Horiz..

[3] Liu Y., Gu H., Jia Y., Liu J., Zhang H., Wang R., Zhang B., Zhang H., Zhang Q. (2019). Design and preparation of biomimetic polydimethylsiloxane (PDMS) films with superhydrophobic, self-healing and drag reduction properties via replication of shark skin and SI-ATRP. Chem. Eng. J..

[4] Descamps L., le Roy D., Tomba C., Deman A. (2021). Magnetic polymers for magnetophoretic separation in microfluidic devices. Magnetochemistry.

[5] Chen C., Shi L.-A., Huang Z., Hu Y., Wu S., Li J., Wu D., Chu J. (2019). Microhole-Arrayed PDMS with Controllable Wettability Gradient by One-Step Femtosecond Laser Drilling for Ultrafast Underwater Bubble Unidirectional Self-Transport. Adv. Mater. Interfaces.

[6] Zhou B., Su B., Ta W., Yang Z., Meng J. (2021). Fabrication of high-aspect-ratio polydimethylsiloxane microstructures by reducing the interfacial adhesion in soft lithography. J. Micromech. Microeng..

[7] Maram S.K., Barron B., Leung J.C.K., Pallapa M., Rezai P. (2018). Fabrication and thermoresistive behavior characterization of three-dimensional silver-polydimethylsiloxane (Ag-PDMS) microbridges in a mini-channel. Sens. Actuators A Phys..

[8] Dabaghi M., Shahriari S., Saraei N., Da K., Chandiramohan A., Selvaganapathy P.R., Hirota J.A. (2021). Surface modification of PDMS-based microfluidic devices with collagen using polydopamine as a spacer to enhance primary human bronchial epithelial cell adhesion. Micromachines.

[9] Raj M.K., Chakraborty S. (2020). PDMS microfluidics: A mini review. J. Appl. Polym. Sci..

[10] Zhou B., Su B., Li M., Meng J. (2020). Microelectroforming of freestanding metallic microcomponents using silver-coated poly (dimethylsiloxane) molds. J. Micromech. Microeng..

[11] Akther F., Yakob S.B., Nguyen N.T., Ta H.T. (2020). Surface Modification Techniques for Endothelial Cell Seeding in PDMS Microfluidic Devices. Biosensors.

[12] Yamashita T., Yasukawa K., Yunoki E. (2019). Fabrication of polydimethylsiloxane (PDMS) fluidic chip using sacrificial template made by fused deposition modeling (FDM) 3D printing and application for flow injection analysis. Anal. Sci..

[13] Liu J., Zheng H., Dai X., Poh P.S., Machens H.G., Schilling A.F. (2020). Transparent PDMS Bioreactors for the Fabrication and Analysis of Multi-Layer Pre-vascularized Hydrogels under Continuous Perfusion. Front. Bioeng. Biotechnol..

[14] Kecili S., Tekin H.C. (2020). Adhesive bonding strategies to fabricate high-strength and transparent 3D printed microfluidic device. Biomicrofluidics.

[15] Faria C.L., Pinho D., Santos J., Gonçalves L.M., Lima R. (2018). Low cost 3D printed biomodels for biofluid mechanics applications. J. Mech. Eng. Biomech..

[16] Chande C., Riaz N., Harbour V., Noor H., Torralba M., Cheng Y.-H., Li Z., Tong A., Voronov R., Basuray S. (2020). Universal method for fabricating PDMS microfluidic device using SU8, 3D printing and soft lithography. Technology.

[17] Chen P.C., Cheng Y.F., Young K.C., Hsieh H.L., Yang C.L. (2016). Design and characterization of a capillary-driven and parallelized microfluidic chip for distributing a liquid plug. Int. J. Precis. Eng. Manuf..

[18] Song K., Gang M.G., Jun M.B.G., Min B.K. (2017). Cryogenic machining of PDMS fluidic channel using shrinkage compensation and surface roughness control. Int. J. Precis. Eng. Manuf..

[19] Zhang G., Sun Y., Liu X., Gao H., Zuo D. (2021). Experimental investigations of machining characteristics on polydimethylsiloxane (PDMS) by cryogenic abrasive air-jet machining. Int. J. Adv. Manuf. Technol..

[20] Chen X., Qu N., Li H., Xu Z. (2016). Electrochemical micromachining of micro-dimple arrays using a polydimethylsiloxane (PDMS) mask. J. Mater. Process. Technol..

[21] Chen X., Qu N., Li H., Zhu D. (2014). The Fabrication and Application of a PDMS Micro Through-Holes Mask in Electrochemical Micromanufacturing. Adv. Mech. Eng..

[22] Isiksacan Z., Guler M.T., Aydogdu B., Bilican I., Elbuken C. (2016). Rapid fabrication of microfluidic PDMS devices from reusable PDMS molds using laser ablation. J. Micromech. Microeng..

[23] Liang C., Su W., Sun X., Hu Y., Duan J.A. (2021). Femtosecond Laser Patterning Wettability-Assisted PDMS for Fabrication of Flexible Silver Nanowires Electrodes. Adv. Mater. Interfaces.

[24] Yong J., Chen F., Huo J., Fang Y., Yang Q., Zhang J., Hou X. (2018). Femtosecond laser induced underwater superaerophilic and superaerophobic PDMS sheets with through microholes for selective passage of air bubbles and further collection of underwater gas. Nanoscale.

[25] Saadat M., Taylor M., Hughes A., Hajiyavand A.M. (2020). Rapid prototyping method for 3D PDMS microfluidic devices using a red femtosecond laser. Adv. Mech. Eng..

[26] Homma K., Watanabe W. (2021). Fabrication of PDMS-based volume Bragg gratings by stitching of femtosecond laser filament. Jpn. J. Appl. Phys..

[27] Ha N.P., Ohishi T., Mizoshiri M. (2021). Direct writing of Cu patterns on polydimethylsiloxane substrates using femtosecond laser pulse-induced reduction of glyoxylic acid copper complex. Micromachines.

[28] Yin J., Chen G., Zhu Z., Jin M., Hu B. (2020). Ablation mechanism investigation and ablation threshold prediction of single crystal diamond irradiated by femtosecond laser. Diam. Relat. Mater..

[29] Huang H., Zhang P., Yu Z., Shen L., Shi H., Tian Y. (2022). Femtosecond laser-induced transformation mechanism from 1D groove structure to 2D microholes structure on the surface of Zr-based metallic glasses. Opt. Laser Technol..

[30] Qu N., Chen X., Li H., Zhu D. (2014). Fabrication of PDMS micro through-holes for electrochemical micromachining. Int. J. Adv. Manuf. Technol..

[31] De Zanet A., Casalegno V., Salvo M. (2021). Laser surface texturing of ceramics and ceramic composite materials–A review. Ceram. Int..

[32] Herman R.M., Wiggins T.A. (1998). Rayleigh range and the M 2 factor for Bessel-Gauss beams. Appl. Opt..

[33] Li Q., Lao H., Lin J., Chen Y., Chem X. (2011). Study of femtosecond ablation on aluminum film with 3D two-temperature model and experimental verifications. Appl. Phys. A.

